# HTLV-1 Proliferation after CD8^+^ Cell Depletion by Monoclonal Anti-CD8 Antibody Administration in Latently HTLV-1-Infected Cynomolgus Macaques

**DOI:** 10.1128/spectrum.01518-23

**Published:** 2023-06-27

**Authors:** Midori Nakamura-Hoshi, Takushi Nomura, Masako Nishizawa, Trang Thi Thu Hau, Hiroyuki Yamamoto, Midori Okazaki, Hiroshi Ishii, Kenzo Yonemitsu, Yuriko Suzaki, Yasushi Ami, Tetsuro Matano

**Affiliations:** a AIDS Research Center, National Institute of Infectious Diseases, Tokyo, Japan; b Joint Research Center for Human Retrovirus Infection, Kumamoto University, Kumamoto, Japan; c Management Department of Biosafety, Laboratory Animal, and Pathogen Bank, National Institute of Infectious Diseases, Tokyo, Japan; d Institute of Medical Science, University of Tokyo, Tokyo, Japan; Kumamoto Daigaku

**Keywords:** CD8^+^ T cell, human T-cell leukemia virus, latent infection, macaque model

## Abstract

Human T-cell leukemia virus type 1 (HTLV-1) induces chronic asymptomatic latent infection with a substantial proviral load but without significant viral replication *in vivo*. Cumulative studies have indicated involvement of CD8-positive (CD8^+^) cells, including virus-specific CD8^+^ T cells in the control of HTLV-1 replication. However, whether HTLV-1 expression from latently infected cells *in vivo* occurs in the absence of CD8^+^ cells remains unclear. Here, we examined the impact of CD8^+^ cell depletion by monoclonal anti-CD8 antibody administration on proviral load in HTLV-1-infected cynomolgus macaques. Five cynomolgus macaques were infected with HTLV-1 by inoculation with HTLV-1-producing cells. Administration of monoclonal anti-CD8 antibody in the chronic phase resulted in complete depletion of peripheral CD8^+^ T cells for approximately 2 months. All five macaques showed an increase in proviral load following CD8^+^ cell depletion, which peaked just before the reappearance of peripheral CD8^+^ T cells. Tax-specific CD8^+^ T-cell responses were detected in these recovered CD8^+^ T cells. Importantly, anti-HTLV-1 antibodies also increased after CD8^+^ cell depletion, indicating HTLV-1 antigen expression. These results provide evidence indicating that HTLV-1 can proliferate from the latent phase in the absence of CD8^+^ cells and suggest that CD8^+^ cells are responsible for the control of HTLV-1 replication.

**IMPORTANCE** HTLV-1 can cause serious diseases such as adult T-cell leukemia (ATL) in humans after chronic asymptomatic latent infection with substantial proviral load. Proviruses are detectable in peripheral lymphocytes in HTLV-1 carriers, and the association of a higher proviral load with a higher risk of disease progression has been observed. However, neither substantial viral structural protein expression nor viral replication was detectable *in vivo*. Cumulative studies have indicated involvement of CD8^+^ cells, including virus-specific CD8^+^ T cells in the control of HTLV-1 replication. In the present study, we showed that CD8^+^ cell depletion by monoclonal anti-CD8 antibody administration results in HTLV-1 expression and an increase in proviral load in HTLV-1-infected cynomolgus macaques. Our results indicate that HTLV-1 can proliferate in the absence of CD8^+^ cells, suggesting that CD8^+^ cells are responsible for the control of HTLV-1 replication. This study provides insights into the mechanism of virus-host immune interaction in latent HTLV-1 infection.

## INTRODUCTION

Human T-cell leukemia virus type 1 (HTLV-1) is a retrovirus that induces chronic asymptomatic infection in humans (1). A small number of HTLV-1-infected carriers develop severe diseases, including adult T-cell leukemia (ATL) and HTLV-1-associated myelopathy or tropical spastic paraparesis (HAM/TSP) after long-term infection (2–7). Proviruses are detectable in peripheral blood mononuclear cells (PBMCs) in HTLV-1 carriers, and the association of higher proviral load with a higher risk of disease progression has been reported (8, 9). However, viral structural protein expression and viral replication were poor *in vivo* (10–12). Viral Tax and structural protein expression is undetectable in freshly isolated PBMCs derived from HTLV-1 infected carriers but becomes detectable after *ex vivo* cell culture (11, 13, 14). Thus, the host has a mechanism to control HTLV-1 replication *in vivo*. Elucidation of the mechanism may contribute to the control of HTLV-1-mediated disease progression.

Cumulative studies have indicated involvement of CD8-positive (CD8^+^) cells, particularly virus-specific CD8^+^ T cells, in the control of HTLV-1 replication (11, 15–17). Some studies have shown an inverse correlation between proviral load and HTLV-1-specific CD8^+^ T-cell responses (18, 19). In particular, HTLV-1 Tax antigen-specific CD8^+^ T-cell responses have been implicated in HTLV-1 control (20–22). However, the extent to which CD8^+^ cells contribute to the control of HTLV-1 replication and whether latently infected cells can begin to induce HTLV-1 proliferation with HTLV-1 expression *in vivo* in the absence of CD8^+^ cells remain unclear.

Macaque models of HTLV-1 or simian T-cell leukemia virus type 1 (STLV-1) infection have been used for analysis of HTLV-1 infection *in vivo* (23–26). These models may not be adequate for analysis of disease progression but can be used for analysis of HTLV-1 control mechanism (27, 28). In the present study, we investigated the effect of CD8^+^ cell depletion on HTLV-1 control in a cynomolgus macaque model of HTLV-1 infection. We used five cynomolgus macaques infected with HTLV-1 by inoculation with HTLV-1-producing cells and performed CD8^+^ cell depletion by administration of these macaques with a monoclonal anti-CD8 antibody. CD8 depletion resulted in increased proviral loads (PVLs) and anti-HTLV-1 antibody levels, indicating that HTLV-1 can proliferate *in vivo* in the absence of CD8^+^ cells.

## RESULTS

### HTLV-1 infection with detectable proviral load in cynomolgus macaques after inoculation with an ATL-derived cell line.

Currently, HTLV-1 infection in cynomolgus macaques by inoculation with an ATL-derived cell line (ATL-040) (29) has been reported by Urano et al. (28). In their study, two cynomolgus macaques intravenously inoculated with 1 × 10^8^ ATL-040 cells showed HTLV-1 infection, with provirus detected in PBMCs, whereas one macaque intravenously inoculated with 1 × 10^7^ ATL-040 cells did not exhibit detectable provirus despite induction of anti-HTLV-1 antibodies.

**FIG 1:**
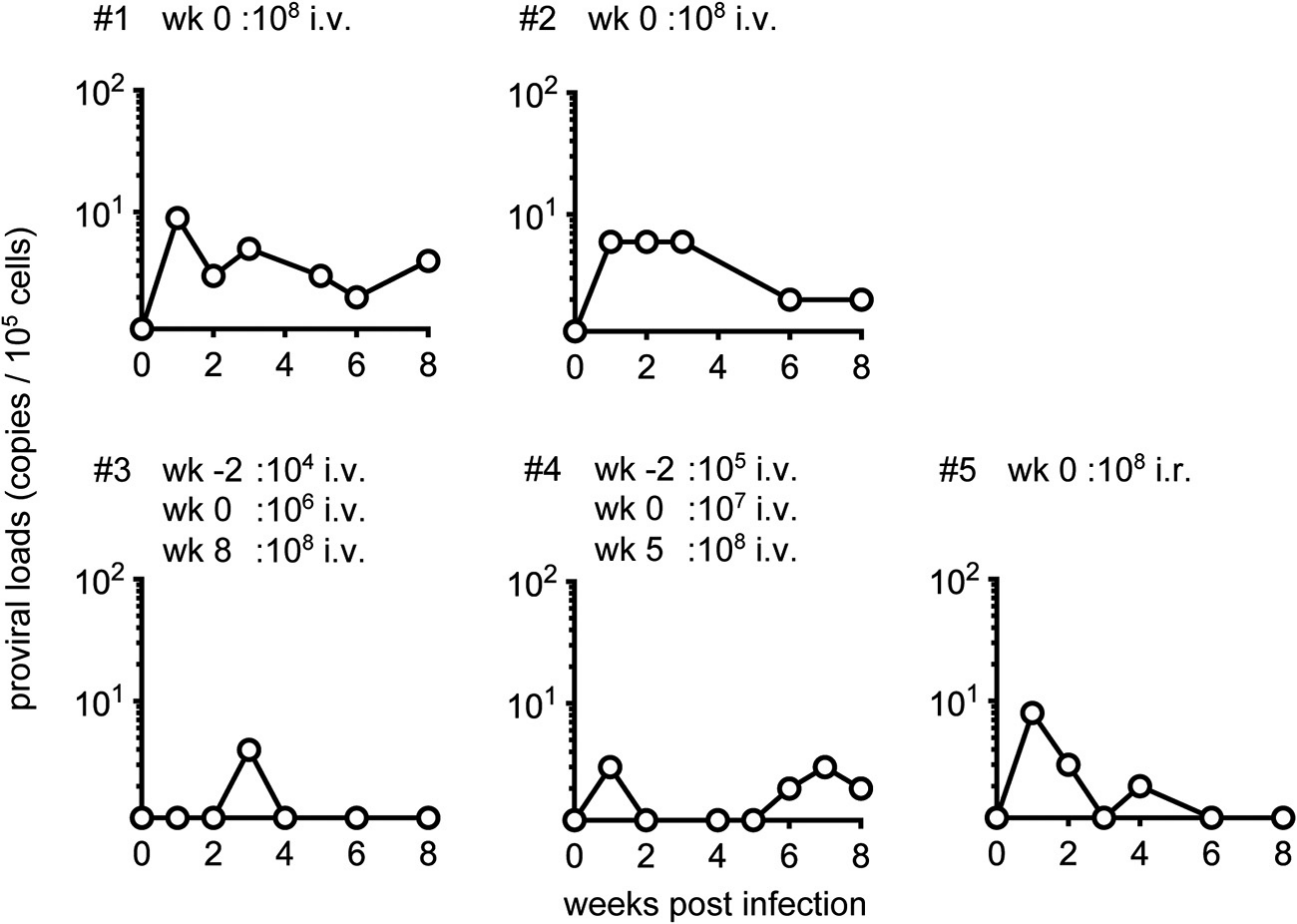
HTLV-1 proviral load in cynomolgus macaques after ATL-040 inoculation. Changes in proviral loads in PBMCs in the acute phase after HTLV-1 infection in macaques 1, 2, 3, 4, and 5 are shown. In macaques 1 and 2, the time point of intravenous inoculation with 10^8^ ATL-040 cells was set as week (wk) 0. In macaques 3 and 4, the time points of intravenous inoculation with 10^6^ and 10^7^ ATL-040 cells were set as wk 0, respectively. In macaque 5, the time point of intrarectal inoculation with 10^8^ ATL-040 cells was set as wk 0.

**FIG 2:**
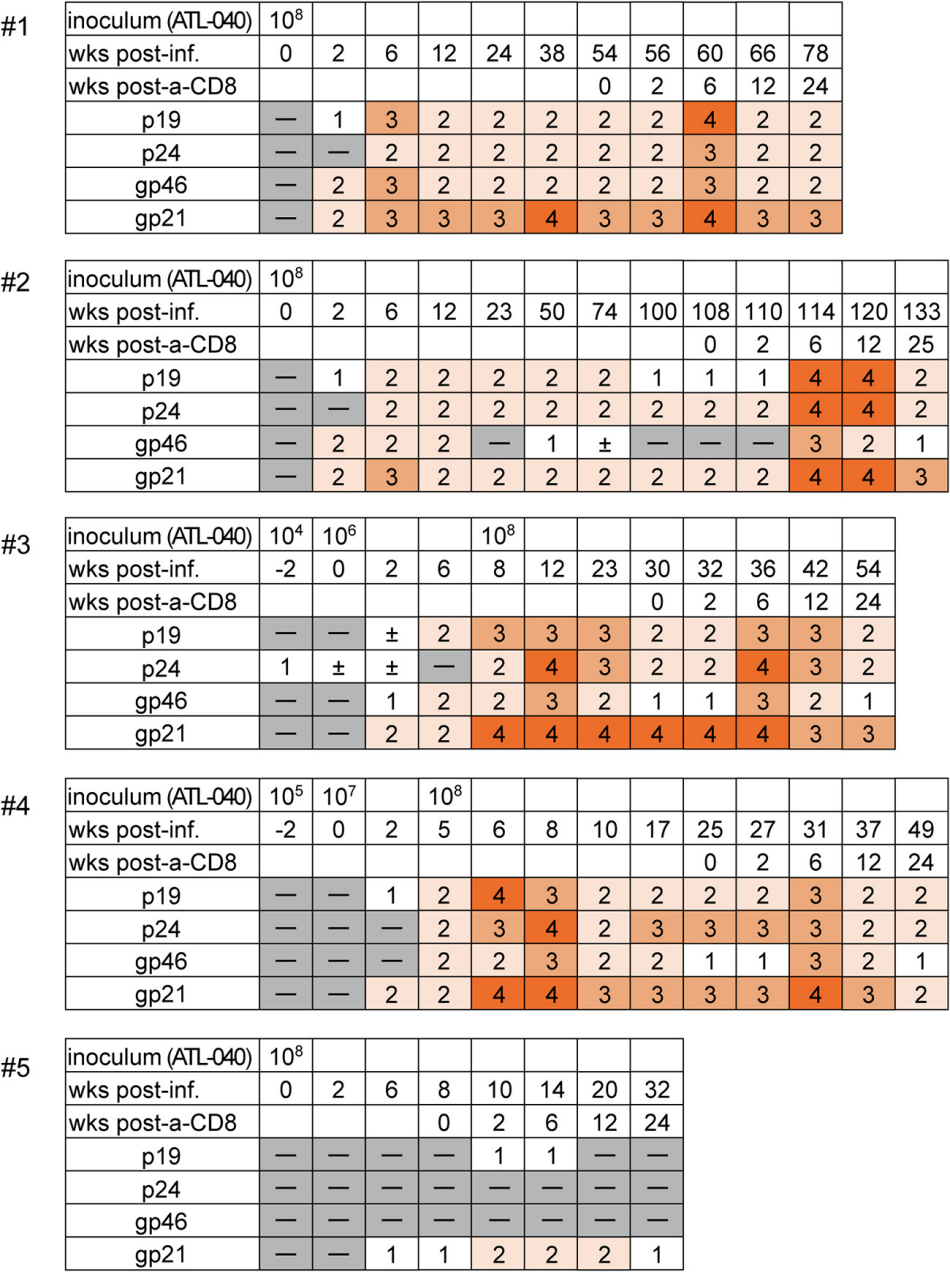
Anti-HTLV-1 antibodies analyzed by line immunoassay in cynomolgus macaques after HTLV-1 infection. The scores for relative levels of plasma anti-HTLV-1 Gag (p19 and p24) and anti-Env (gp46 and gp21) antibodies were determined based on the band (line) intensity in the line immunoassay. Macaque 3 exhibiting positive only for the p24-specific line at week −2 was considered HTLV-1 naive before ATL-040 inoculation according to the INNO-LIA instructions. Monoclonal anti-CD8 antibody was administered three times (days 0, 3, and 7) starting from week 54 in macaque 1, week 108 in macaque 2, week 30 in macaque 3, week 25 in macaque 4, and week 8 in macaque 5 postinfection, respectively. Scoring was as follows: −, negative; ±, marginal. Scores 1 through 4 are positive, with 1 being the lowest and 4 the highest levels.

In the present study, we first intravenously inoculated two cynomolgus macaques 1 and 2 with 1 × 10^8^ ATL-040 cells. Persistent HTLV-1 infection was confirmed in both macaques by detection of proviruses in PBMCs at week 1 after ATL-040 inoculation and later (for at least 2 months) (Fig. 1). HTLV-1 protein-specific antibodies, measured by the line immunoassay (INNO-LIA HTLV I/II; Fujirebio), were detected at week 2 postinfection (Fig. 2).

Second, we attempted intravenous inoculation with lower doses of ATL-040 cells. Macaques 3 and 4 were intravenously inoculated with 1 × 10^4^ and 1 × 10^5^ ATL-040 cells, respectively, but proviruses in PBMCs were undetectable at week 1 after the ATL-040 inoculation (Fig. 1). These macaques were then intravenously inoculated with 1 × 10^6^ and 1 × 10^7^ ATL-040 cells, respectively, 2 weeks after the first inoculation. Proviruses in PBMCs were undetectable just before the second ATL-040 inoculation but detected at weeks 3 and 1 after the second inoculation in macaques 3 and 4, respectively (Fig. 1). Anti-HTLV-1 antibodies were undetectable at week 0 but became detectable at week 2 after the second inoculation in both of the animals (Fig. 2). Thus, these two macaques were considered to be infected with HTLV-1 at least by the second ATL-040 inoculation. However, proviruses were undetectable after that. Then, macaques 3 and 4 were intravenously inoculated with 1 × 10^8^ ATL-040 cells at weeks 8 and 5 postinfection (after the second inoculation), respectively. Proviruses remained undetectable in macaque 3, while macaque 4 showed persistent detectable PVLs after the third inoculation (Fig. 1).

Third, we attempted intrarectal inoculation with ATL-040 cells. Proviruses in PBMCs became detectable in macaque 5 after intrarectal inoculation with 1 × 10^8^ ATL-040 cells (Fig. 1). The line immunoassay showed induction of detectable anti-HTLV-1 antibodies not at week 2 but at week 6 after ATL-040 inoculation in this macaque (Fig. 2).

### Increase in PVLs by CD8^+^ cell depletion in the chronic phase of HTLV-1 infection.

**FIG 3:**
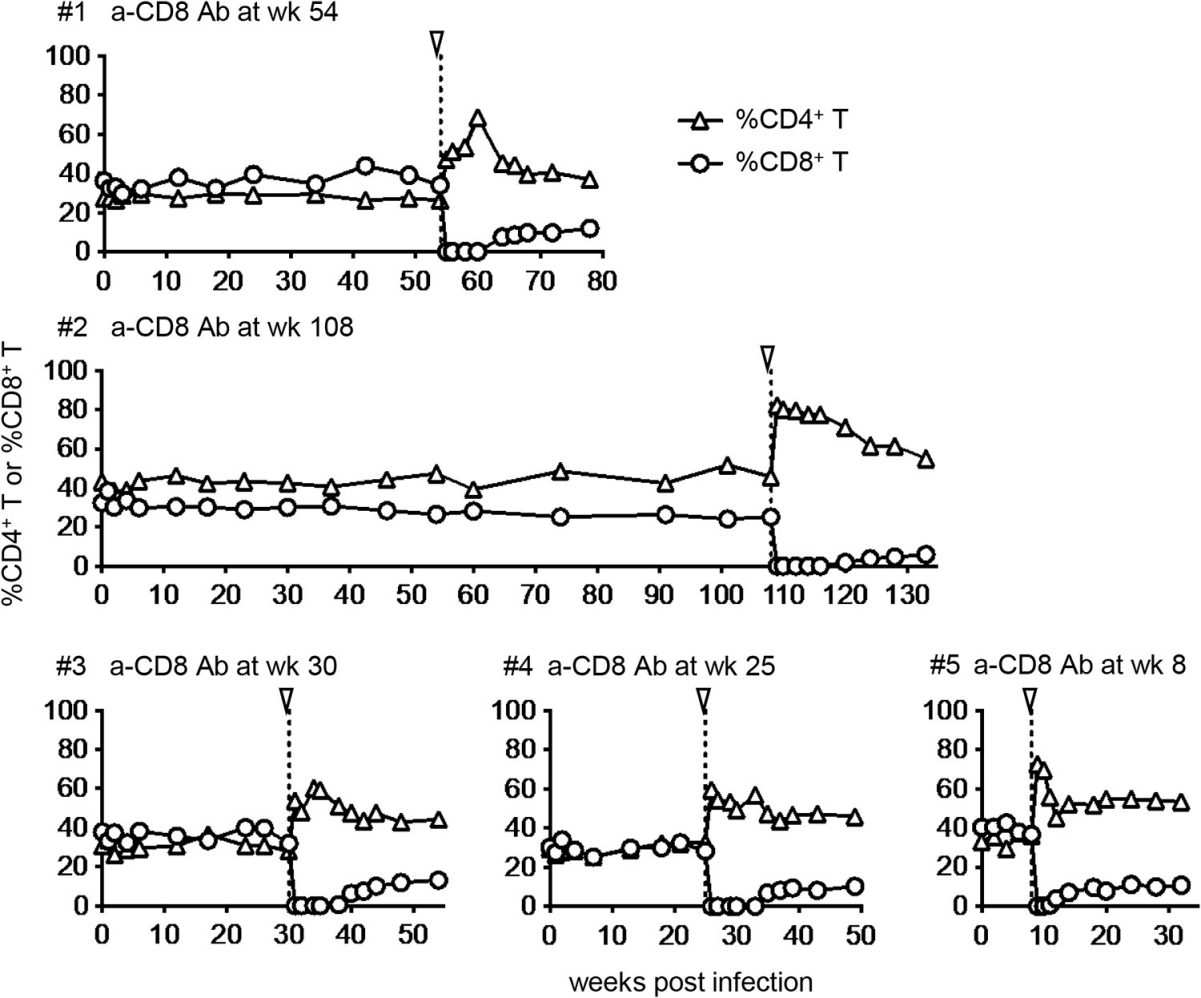
Peripheral blood CD4^+^ T and CD8^+^ T-cell frequencies in cynomolgus macaques after HTLV-1 infection. Changes in CD3^+^ CD4^+^ T (open triangles) and CD3^+^ CD8^+^ (open circles) cell frequencies in PBMCs after HTLV-1 infection. These cells do not include CD3^+^ CD4^+^ CD8^+^ cells, which were also depleted by anti-CD8 antibody administration. The arrowheads indicate the time point of initial anti-CD8 antibody administration.

In these five HTLV-1-infected cynomolgus macaques described above, we performed CD8^+^ cell depletion by administration of a monoclonal anti-CD8 antibody, MT807-R1, in the chronic phase of infection. The anti-CD8 antibody was administered three times (days 0, 3, and 7) at different time points postinfection. CD8^+^ T cells in PBMCs became undetectable 3 days after the first anti-CD8 antibody administration and remained undetectable for 6 weeks or longer, except for macaque 5, with marginal CD8^+^ T cell reappearance after 4 weeks (Fig. 3). These animals exhibited similar patterns of CD3^−^ CD8^+^ cell depletion in PBMCs after anti-CD8 antibody administration (see Fig. S1 in the supplemental material).

**FIG 4:**
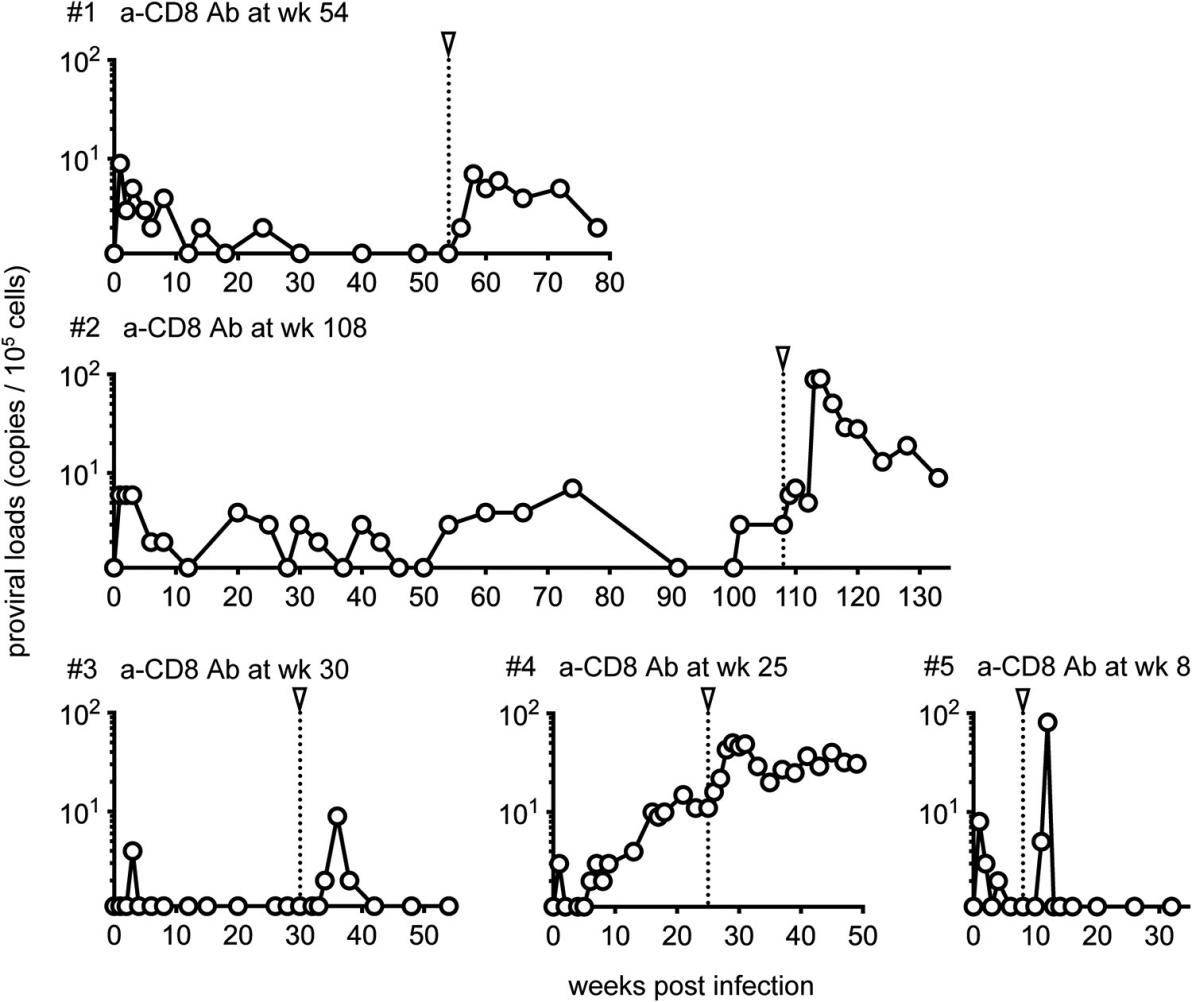
HTLV-1 proviral load in cynomolgus macaques after anti-CD8 antibody administration. Changes in proviral loads in PBMCs after HTLV-1 infection in macaques 1, 2, 3, 4, and 5 are shown. The arrowheads indicate the time point of initial anti-CD8 antibody administration.

In macaques 1, 3, and 5, proviruses in PBMCs were undetectable just before the anti-CD8 antibody administration but became detectable after the antibody administration (Fig. 4). In macaques 2 and 5, PVLs increased after the anti-CD8 antibody administration (Fig. 4). In macaque 1, PVLs became detectable at 2 weeks and peaked at 4 to 6 weeks after the initial anti-CD8 antibody administration (at week 54 postinfection) (Fig. 4). In macaque 2, PVLs began to increase at 1 week and peaked at 5 to 6 weeks after the initial anti-CD8 antibody administration (at week 108 postinfection). In macaque 3, PVLs became detectable at 4 weeks and peaked at 6 weeks after the initial anti-CD8 antibody administration (at week 30 postinfection). In macaque 4, PVLs began to increase at 1 week and peaked at 4 to 6 weeks after the initial anti-CD8 antibody administration (at week 25 postinfection). In macaque 5, PVLs became detectable at 3 weeks and peaked at 4 weeks after the initial anti-CD8 antibody administration (at week 8 postinfection) and then became undetectable with detectable peripheral CD8^+^ T cells. Thus, in macaques 1, 3, and 5, proviruses in PBMCs were undetectable just before anti-CD8 antibody administration but became detectable following antibody treatment. In macaques 2 and 4, PVLs increased after anti-CD8 antibody administration (Fig. 4). In all five macaques, anti-CD8 antibody administration resulted in CD8^+^ cell depletion with an increase (more than 4-fold) in PVLs, peaking before the recovery of peripheral CD8^+^ T cells.

### Increase in anti-HTLV-1 antibodies by CD8^+^ cell depletion in the chronic phase of HTLV-1 infection.

The line immunoassay allows scoring of relative levels of plasma anti-HTLV-1 Gag (p19 and p24) and anti-Env (gp46 and gp21) antibodies. In all five macaques, the anti-HTLV-1 Gag and the anti-Env scores increased and peaked approximately at week 6 after anti-CD8 antibody administration (Fig. 2).

**FIG 5:**
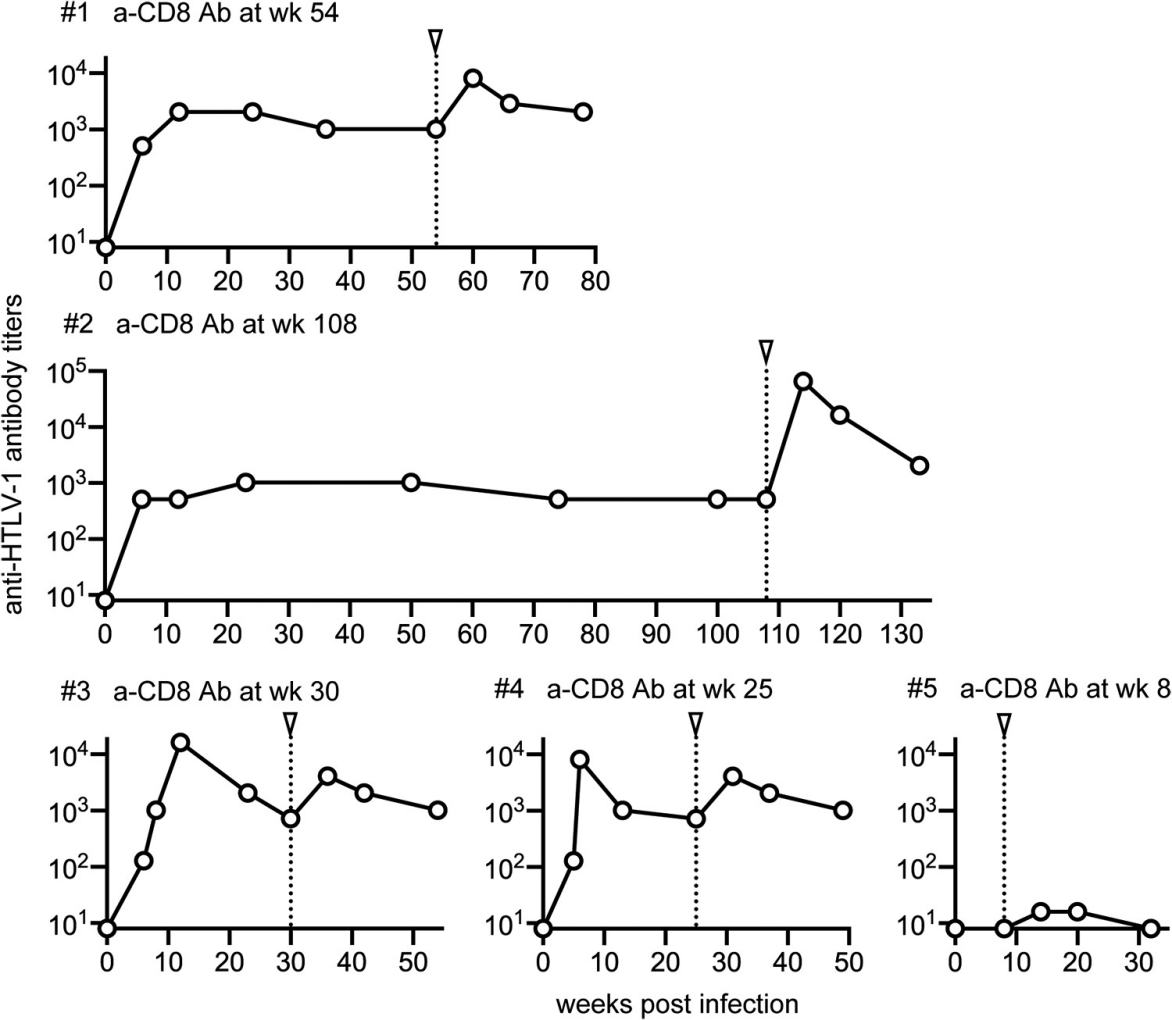
Anti-HTLV-1 antibodies analyzed by particle agglutination assay after HTLV-1 infection. Changes in anti-HTLV-1 antibody titers measured by particle agglutination assay after HTLV-1 infection are shown.

To confirm the increase in anti-HTLV-1 antibodies by CD8^+^ cell depletion, we examined plasma anti-HTLV-1 antibody levels by particle agglutination assay using the Serodia kit. All macaques showed an increase in anti-HTLV-1 antibody levels, peaking at approximately week 6 after anti-CD8 antibody administration, though this increase was less pronounced in macaque 5 (Fig. 5). Anti-HTLV-1 antibody levels were lower in macaque 5 prior to anti-CD8 antibody administration, which may be due to the difference in the challenge route. Additionally, peripheral CD8^+^ T cells reappeared earlier after anti-CD8 antibody administration for macaque 5. These may account for the lower increase in anti-HTLV-1 antibodies observed following CD8^+^ cell depletion in macaque 5.

**FIG 6:**
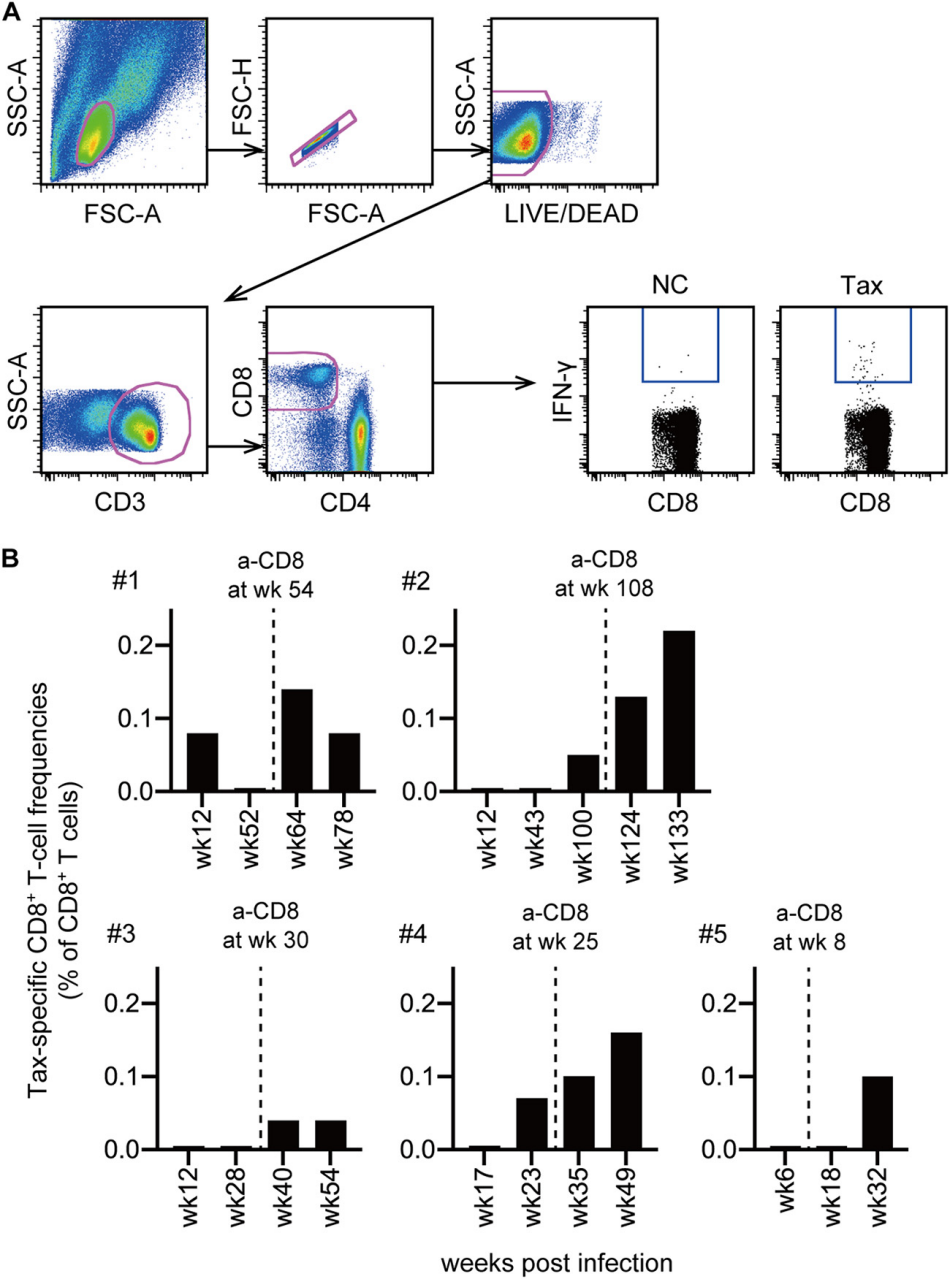
Tax-specific CD8^+^ T-cell responses in cynomolgus macaques after HTLV-1 infection. (A) Representative gating schema for detection of specific IFN-γ induction after stimulation in flow cytometric analysis. Data on PBMCs of macaque 2 at week 133 without stimulation (NC) and with stimulation using overlapping peptides spanning Tax amino acids 1 to 125 (Tax) are shown. (B) Tax-specific CD8^+^ T-cell frequencies in PBMCs before and after CD8^+^ cell depletion are shown.

Finally, we investigated HTLV-1 Tax-specific CD8^+^ T-cell responses by flow cytometric analysis of interferon gamma (IFN-γ) induction after specific stimulation (Fig. 6). Prior to anti-CD8 antibody administration, Tax-specific CD8^+^ T-cell responses were detected in three macaques (1, 2, and 4), all of which showed persistent detectable proviruses, but they were undetectable in the remaining two macaques (3 and 5) with detectable provirus only in the acute phase of infection. After recovery of peripheral CD8^+^ T cells following CD8^+^ cell depletion, Tax-specific CD8^+^ T-cell responses were induced in all five macaques (Fig. 6).

## DISCUSSION

CD8^+^ cells, in particular, CD8^+^ T cells, have been indicated to play a role in the control of HTLV-1 replication *in vivo* (11, 15–17). However, whether HTLV-1 can proliferate in the chronic phase in the absence of CD8^+^ cells has remained unclear. In the previous study by Urano et al. (28), the effect of anti-CD8 antibody administration was examined in HTLV-1-infected macaques. However, the number of animals was limited (*n* = 2), and the increase in PVLs after antibody administration was marginal in one of the two, which may possibly be because the animals received only one injection of anti-CD8 antibody, resulting in a shorter period of CD8^+^ cell depletion. Therefore, in the present study, we performed CD8^+^ cell depletion by three times of anti-CD8 antibody administration in the chronic phase of HTLV-1 infection by using a larger number of macaques to obtain evidence demonstrating that HTLV-1 can proliferate *in vivo* in the absence of CD8^+^ cells.

HTLV-1 proliferation can possibly occur by proliferation of latently HTLV-1-infected cells or by HTLV-1 replication with cell-to-cell HTLV-1 transmission from infected cells to uninfected cells. Our results showing an increase in anti-HTLV-1 antibodies indicate HTLV-1 antigen expression, implying the latter mechanism, HTLV-1 replication by anti-CD8 antibody administration inducing CD8^+^ cell depletion. This HTLV-1 replication is considered to occur because of CD8^+^ cell depletion, implying a crucial role of CD8^+^ cells for the control of HTLV-1 replication *in vivo*, while possible activation of HTLV-1-infected T cells by antibody administration may also be involved in enhancement of HTLV-1 expression (30–32).

In HTLV-1-infected carriers, viral Tax and structural protein expression is poor in latently HTLV-1-infected cells. These cells are known to express HTLV-1 HBZ, but not efficiently (33–35). Thus, these latently HTLV-1-infected cells poorly expressing viral antigens are not eliminated even in the presence of HTLV-1-specific CD8^+^ T cells, resulting in persistent PVLs in HTLV-1 carriers. However, it has been suggested that some HTLV-1-infected cells transiently express Tax (36, 37). It is speculated from our results that these Tax-expressing cells are eliminated by CD8^+^ T cells, leading to suppression of HTLV-1 proliferation from these cells. This CD8^+^ cell depletion model may be useful for analysis of the interaction between host immune and HTLV-1 antigen-expressing cells. Anti-CD8 antibody administration depleted not only CD8^+^ T cells but also CD3^−^ CD8^+^ cells such as NK cells (see Fig. S1 in the supplemental material). Involvement of NK cells in primary HTLV-1 infection has been suggested in macaques (38). Thus, in addition to HTLV-1-specific CD8^+^ T cells, NK cells may be involved in HTLV-1 control during the chronic phase of infection.

In our cynomolgus macaque model of HTLV-1 infection, intravenous inoculation with 1 × 10^8^ ATL-040 cells resulted in HTLV-1 infection with detectable proviruses at week 1 postinoculation. On the other hand, proviruses were undetectable, and HTLV-1 infection was not confirmed after intravenous inoculation with 1 × 10^4^ or 1 × 10^5^ ATL-040 cells. Intravenous inoculation with 1 × 10^6^ or 1 × 10^7^ ATL-040 cells resulted in HTLV-1 infection with detectable proviruses. Thus, it is speculated that HTLV-1 infection can be consistently established by intravenous inoculation with 1 × 10^8^ ATL-040 cells, while intravenous inoculation with 1 × 10^6^ or 1 × 10^7^ ATL-040 cells may be sufficient for the establishment of HTLV-1 infection. Induction of anti-HTLV-1 antibodies was observed at week 2 after inoculation with 1 × 10^6^ to 1 × 10^8^ ATL-040 cells. However, there is a possibility that detectable anti-HTLV-1 antibody responses can be induced only by inoculums without infection, and it remains unclear whether this could be an indicator of the establishment of HTLV-1 infection.

Currently, an increase in HTLV-1 infection via sexual transmission has been indicated (39–41). We thus attempted intrarectal inoculation with HTLV-1-producing cells into one macaque. Our attempt, for the first time, confirmed the establishment of HTLV-1 infection by intrarectal ATL-040 inoculation although PVLs were low. This could be a new model for HTLV-1 sexual transmission in the future.

In summary, the present study shows that anti-CD8 antibody administration inducing CD8^+^ cell depletion in the chronic phase of HTLV-1 infection results in an increase in PVLs and anti-HTLV-1 antibody levels in cynomolgus macaques. This result provides evidence indicating that HTLV-1 can proliferate from the latent phase in the absence of CD8^+^ cells, suggesting that CD8^+^ cells are responsible for the control of HTLV-1 replication *in vivo*.

## MATERIALS AND METHODS

### Ethics statement.

Animal experiments using cynomolgus macaques (Macaca fascicularis) were performed at the National Institute of Infectious Diseases (NIID) after approval by the Committee on the Ethics of Animal Experiments in NIID (permission number 517006) under the guidelines for animal experiments in accordance with the Guidelines for Proper Conduct of Animal Experiments established by the Science Council of Japan (http://www.scj.go.jp/ja/info/kohyo/pdf/kohyo-20-k16-2e.pdf). The experiments were in accordance with the Weatherall report for the use of nonhuman primates in research recommendations (https://royalsociety.org/topics-policy/publications/2006/weatherall-report/). Each macaque was housed in a separate cage and received standard primate feed and fresh fruit daily. Virus inoculation, blood collection, and anti-CD8 antibody treatment were performed under ketamine anesthesia.

### Animal experiments.

Cynomolgus macaques were intravenously or intrarectally inoculated with ATL-040 cells for HTLV-1 infection. ATL-040 is an ATL-derived cell line producing HTLV-1 (kindly provided by Yuetsu Tanaka [29]). For CD8^+^ cell depletion, macaques were administered with 5 mg/kg body weight of anti-CD8α monoclonal antibody clone MT807-R1 (NIH Nonhuman Primate Reagent Resource) three times on days 0, 3, and 7 after the first anti-CD8 antibody administration.

### Flow cytometric analysis of cell surface markers.

Whole-blood samples were treated with lysing solution (BD) and subjected to surface staining using anti-CD3 allophycocyanin (APC) (clone SP34-2; BD), anti-CD4 fluorescein isothiocyanate (FITC) (clone M-T477; BD), anti-CD8 PerCP (clone SK1; BD), and anti-CD20 phycoerythrin (PE) (clone 2H7; BD) antibodies. Alternatively, whole-blood samples from anti-CD8 antibody-treated animals were stained with anti-CD3 APC, anti-CD4 PerCP (clone L200; BD), anti-CD8 FITC (clone DK25; FujiFilm), and anti-CD20 PE. The anti-CD8 (clone DK25) antibody can recognize CD8 without competing with MT807-R1. Stained cells were analyzed by BD FACSCanto II with FACSDiva v8.0.1 (BD) and FlowJo v9.2 (FlowJo LLC). For gating of CD3^+^ CD8^+^ and CD3^−^ CD8^+^ cell subsets, singlet cells were gated from PBMC subsets, followed by gating of CD20^−^ subsets. Then, CD3^+^ CD8^+^ and CD3^−^ CD8^+^ subsets were determined in CD3-CD8 dot plots, respectively.

### Quantification of HTLV-1 *tax* proviral load.

Cellular DNA extracted from PBMCs was used for the measurement of *tax* proviral load. PBMCs were prepared from whole blood by density gradient centrifugation using Ficoll-Paque Plus (Cytiva). Cellular DNA was extracted from 5 × 10^6^ of PBMCs using Qiagen DNeasy blood and tissue kit (Qiagen) and subjected to real-time PCR for HTLV-1 *tax* DNA quantification using TaqMan Fast Universal master mix (Thermo Fisher Scientific) and QuantStudio 5 (Thermo Fisher Scientific) according to manufacturers’ protocols. The primers pX2-S (5′-CGGATACCCAGTCTACGTGTT-3′) and pX2-AS (5′-CAGTAGGGCGTGACGATGTA-3′) and pX2-probe (5′-FAM-CTGTGTACAAGGCGACTGGTGCC-TAMRA-3′) were used for detecting *tax* proviruses (42). The RNase P gene was quantified as an internal control using TaqMan copy number reference assay (Thermo Fisher Scientific). Cellular DNA of TL-Om1 cells (an ATL-derived cell line kindly provided by Isao Hamaguchi [43]) was extracted and used as a standard DNA for quantification of *tax* and RNase P. *tax* and RNase P copy numbers in each sample were calculated using standard curves based on threshold cycle (*C_T_*) values of serially diluted TL-Om1 DNAs. The lower limit of detection is 2 copies/10^5^ cells.

### Analysis of anti-HTLV-1 antibodies.

Anti-HTLV-1 antibodies were examined by line immunoassay (INNO-LIA HTLV I/II; Fujirebio) and by particle agglutination assay (Serodia HTLV-I; Fujirebio). Plasma samples were heat inactivated at 56°C for 30 min. In INNO-LIA, 10 μL of heat-inactivated plasma samples were dispensed to 1 mL of kit-provided diluent and incubated with HTLV-1 antigen-coated strip overnight, followed by incubation with kit-provided conjugate solution and substrate solution. Then, the reaction was stopped, and the scores for relative levels of antibodies specific for individual antigens (Gag [p19 and p24] and Env [gp46 and gp21]) were determined based on the band (line) intensity according to the manufacturer's instructions. In Serodia, heat-inactivated plasma samples were serially diluted with kit-provided diluent and mixed with sensitized particles. Two hours later, the patterns of particles were evaluated. Endpoint titers were determined according to the manufacturer’s instructions. The lower limit of detection is a 1:16 dilution.

### Analysis of Tax-specific CD8^+^ T-cell responses.

HTLV-1 Tax-specific CD8^+^ T-cell frequencies were measured by flow cytometric analysis of gamma interferon (IFN-γ) induction after specific stimulation as described previously (44). Briefly, PBMCs were cocultured with autologous herpesvirus papio-immortalized B-lymphoblastoid cell lines (B-LCL) pulsed with peptide pools (at a final concentration of 2 μM for each peptide) using panels of overlapping peptides spanning HTLV-1 Tax amino acid sequences in the presence of GolgiStop (monensin; BD) for 6 h. Intracellular IFN-γ staining was performed using a Cytofix/Cytoperm kit (BD) and Live/Dead fixable aqua dead cell stain kit (Invitrogen), anti-CD3 APC-Cy7 (clone SP34-2; BD), anti-CD4 FITC, anti-CD8 PerCP, and anti-IFN-γ PE (clone 4S.B3; BioLegend). Stained cells were analyzed by BD FACSCanto II with FACSDiva v8.0.1 and FlowJo v9.2. Specific CD8^+^ T-cell frequencies were calculated by subtracting nonspecific IFN-γ^+^ CD8^+^ T-cell frequencies from those after peptide-specific stimulation. Specific CD8^+^ T-cell frequencies less than 0.02% of CD8^+^ T cells were considered negative.

### Data availability.

All data supporting the conclusion in this study are included in the main text and figures, and additional information is available from the corresponding author upon request.
