# Supplementary material for: HTLV-1 Proliferation after CD8+ Cell Depletion by Monoclonal Anti-CD8 Antibody Administration in Latently HTLV-1-Infected Cynomolgus Macaques
Source: Microbiol Spectr. 2023 Jun 27;11(4):e01518-23. doi: 10.1128/spectrum.01518-23 (PMC10434050; doi:10.1128/spectrum.01518-23)
Supplement: Supplemental file 1 — Supplemental material. Download spectrum.01518-23-s0001.pdf, PDF file, 0.1 MB [file spectrum.01518-23-s0001.pdf]

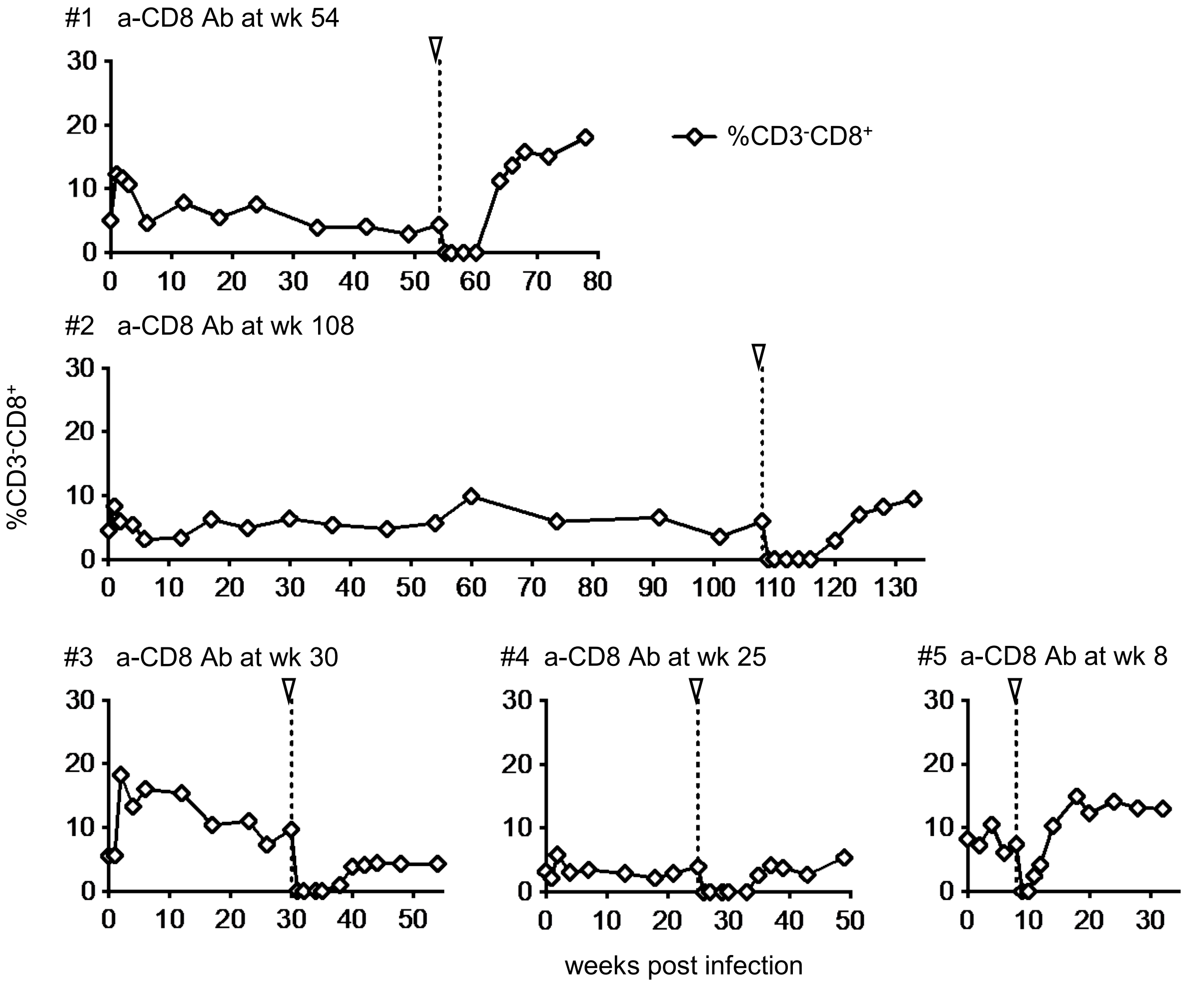

Fig. S1. Peripheral blood CD3$^-$CD8$^+$ cell frequencies in cynomolgus macaques after HTLV-1 infection.

Changes in CD3$^-$CD8$^+$ cell frequencies in PBMCs after HTLV-1 infection. The arrowheads indicate the time point of initial anti-CD8 antibody administration.
